# Prenatal diagnosis of sirenomelia in the second trimester of pregnancy using two-dimensional ultrasound, three-dimensional ultrasound and magnetic resonance imaging

**DOI:** 10.1590/0100-3984.2015.0212

**Published:** 2017

**Authors:** Heron Werner, Pedro Daltro, Tatiana Fazecas, Bianca Ribeiro, Edward Araujo Júnior

**Affiliations:** 1 Clínica de Diagnóstico por Imagem (CPDI), Rio de Janeiro, RJ, Brazil.; 2 Escola Paulista de Medicina da Universidade Federal de São Paulo (EPM-Unifesp), São Paulo, SP, Brazil.

**Figure 1:**
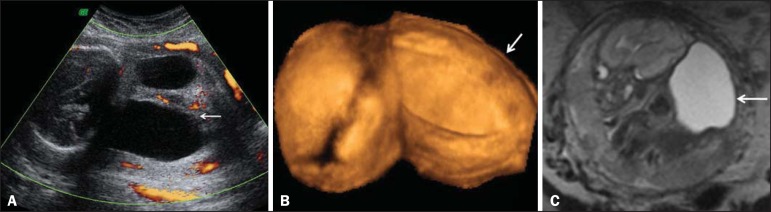
Prenatal findings of sirenomelia at 26 weeks and 5 days of gestation: 2D ultrasound with color Doppler in the axial plane shows myelomeningocele. Note that the mass is very close to the neck (arrow, **A**); same view at 3D ultrasound in the rendering mode (**B**), and at T2-weighted MRI sequence in the sagittal plane (**C**). Note that the mass of lumbar origin (myelomeningocele) is very close to the cervical region of the fetus (arrow, **C**).

**Figure 2:**
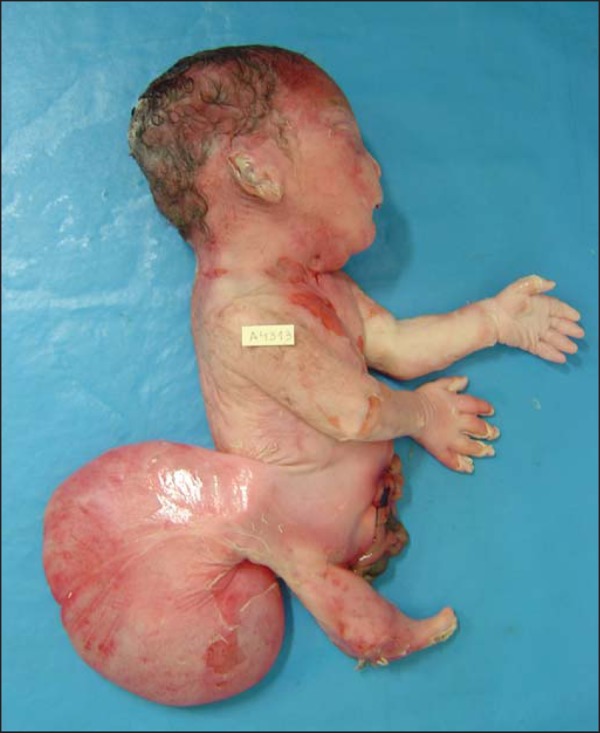
Postmortem evaluation of a 29-week stillborn fetus with sirenomelia.

Dear Editor,

A 30-year-old woman was referred at 23 weeks of gestation due to olygohydramnios, together with short fetal femur length and cystic hygroma. It was the first pregnancy for a non-consanguineous couple with a family history of neural tube defects. The patient reported chronic arterial hypertension during her pregnancy. The previous ultrasound findings were confirmed at our facility. Two-dimensional (2D) ultrasound showed fusion of the lower limbs, and color Doppler ultrasound revealed no vascularization of the lower limbs (Figure 1A). Three-dimensional (3D) ultrasound in the rendering mode confirmed the findings of the 2D ultrasound (Figure 1B). For a better understanding of the fetal morphology due to the olygohydramnios, magnetic resonance imaging (MRI) was performed. The MRI scan showed myelomeningocele and bilateral renal agenesis, as well as showing no identifiable characteristics of the lower limbs (Figure 1C). Termination of the pregnancy was authorized at 29 weeks of gestation. The stillborn infant weighed 1120 g. Pathologic investigation showed sirenomelia (*sympus apus*), lumbar myelomeningocele, and interventricular communication (Figure 2). Radiographic studies showed only one femur (sirenomelia type VII according to the Stocker and Heifetz classification).

Sirenomelia is a rare congenital anomaly with an estimated incidence of 1:60,000 live births^(1)^. It is defined by fused lower limbs, a single umbilical artery, and genitourinary anomalies^(2)^. In approximately 25-30% of cases, sirenomelia is accompanied by other congenital anomalies, such as congenital heart disease and gastrointestinal anomalies^(1)^. The prenatal diagnosis is based on identification of this pattern of malformation in imaging studies.

Sirenomelia is considered a primary developmental field defect affecting multiple midline primordia^(3)^. In the case reported here, MRI allowed us to make the diagnosis of myelomeningocele, which was identified as cystic hygroma on prenatal ultrasound, and bilateral renal agenesis, thereby confirming severe fetal impairment, which allowed the termination of pregnancy to be authorized. However, not all of the associated malformations were identified prior to the stillbirth; the interventricular communication and gastroschisis were identified only during the autopsy. Congenital heart disease has been associated with sirenomelia^(1,4)^, and the fetus evaluated here was also exposed to angiotensin-converting enzyme inhibitors, which could also explain the occurrence of the cardiac defect^(5)^.

The combination of interventricular communication and gastroschisis is not very common; in fact, only two cases, both identified by prenatal ultrasound, have been reported^(6)^. In a recent review, Feldkamp et al.^(7)^ suggested that gastroschisis is a primary malformation. Our case showed the importance of using a combination of different imaging methods for the diagnosis of a rare congenital anomaly. Although ultrasound continues to be the main diagnostic tool for use during pregnancy, MRI has many advantages, mainly in identifying the fetal morphology^(8)^. In the case presented here, despite the high quality of the images, the associated malformations were identified only through pathological studies. The unusual anomalies identified in this case were defects of blastogenesis. The combination of prenatal imaging and postnatal autopsy is important to defining the spectrum of associated malformations even when the congenital anomaly is part of a primary developmental field defect.
